# Internet of things-based home noninvasive ventilation in COPD patients with hypercapnic chronic respiratory failure: study protocol for a randomized controlled trial

**DOI:** 10.1186/s13063-022-06372-z

**Published:** 2022-05-12

**Authors:** Weipeng Jiang, Yuanlin Song

**Affiliations:** 1 Department of Pulmonary Medicine, Zhongshan Hospital, Fudan University, Shanghai, 200032 China; 2 Shanghai Respiratory Research Institute, Shanghai, 200032 China; 3 National Clinical Research Center for Aging and Medicine, Huashan Hospital, Fudan University, Shanghai, 200000 China; 4 Department of Pulmonary Medicine, Qingpu Branch, Zhongshan Hospital, Fudan University, Shanghai, 201700 China; 5 Jinshan Hospital of Fudan University, Shanghai, 201508 China

**Keywords:** Chronic obstructive pulmonary disease, Noninvasive positive pressure ventilation, Hypercapnia respiratory failure, Internet of things, Tele-monitoring, Follow-up, Management

## Abstract

**Background:**

Home noninvasive positive pressure ventilation (NIPPV) has become evidence-based care for stable hypercapnic chronic obstructive pulmonary disease (COPD) patients. There are still other challenges including appropriate follow-up, telemonitor, and management to ensure treatment effectiveness, compliance, and security and to improve quality of life. The Internet of things (IOT) is the name given to the network of devices and other “things” with built-in sensors, software, electronics, and network connectivity, communicating these objects over wireless networks and sending data to a cloud platform. The study aims to evaluate the effectiveness and safety of the IOT-based management of NIPPV for the COPD patients with hypercapnic chronic respiratory failure.

**Methods:**

This multicenter, prospective, randomized controlled trial was conducted with a total of 200 COPD patients with chronic hypercapnic respiratory failure. Using a computer-generated randomization process, patients were randomized (in a 1:1 ratio) into the usual NIPPV (control group) or to receive additional IOT-based management (intervention group) for 12 months. The primary outcome was the Severe Respiratory Insufficiency (SRI) questionnaire. Secondary outcomes included compliance with the ventilator, gas exchange, lung function, health-related quality of life, hospitalization frequency, time to death within 1-year, all-cause mortality, safety analysis, and cost-effectiveness analysis.

**Discussion:**

This study will be the first and largest randomized trial in China to evaluate the effectiveness and safety of the IOT-based management of NIPPV for COPD patients with chronic hypercapnic respiratory failure. The results will help to understand the current situation of IOT-based home ventilation and may provide new evidence for home NIPPV treatment and management in the future.

**Trial registration:**

Chinese Clinical Trials Registry ChiCTR1800019536. Registered on 17 November 2018.

**Supplementary Information:**

The online version contains supplementary material available at 10.1186/s13063-022-06372-z.

## Background

Home non-invasive positive pressure ventilation (NIPPV) has long been undetermined in patients with stable chronic obstructive pulmonary disease (COPD) with chronic hypercapnic respiratory failure (CHRF) [1–7]. Previous research yielded conflicting results on survival, re-hospitalization, lung function, gas exchange, exercise tolerance, and quality of life [3–8]. However, recent randomized controlled trials have indicated that high-intensity NIPPV, which used high levels of inspiratory positive airway pressure (IPAP) with a high backup respiratory rate aimed at maximal PaCO2 reduction, showed physiological and clinical benefits [9, 10]. On the basis of these positive results, a recent meta-analysis demonstrated that home NIPPV was associated with a lower risk of mortality and all-cause re-hospitalization compared with no device support, but no significant difference in quality of life had been identified [11]. NIPPV has become evidence-based care for stable hypercapnic COPD patients [12–14].

There are still other challenges including appropriate follow-up, telemonitor, and management to ensure treatment effectiveness, adherence, and security and to improve quality of life. Usually, hospitals prescribe NIPPV, companies sold the ventilator while patients have little information of knowing how to use and maintain NIPPV at home [15, 16]. In fact, actively monitoring, communicating information, and solving problems during follow-up were crucial for long-term adherence and clinical outcomes [1, 16–18]. Furthermore, home NIPPV can be considered not only as a treatment, but also as a tele-monitoring predictor of objectively detecting acute exacerbations of COPD onset [16, 18]. However, the increasing number and costs of ventilator-dependent individuals make health providers largely insufficient to face the demands.

The Internet of things (IOT) is the name given to the network of devices and other “things” with built-in sensors, software, electronics, and network connectivity, communicating these objects over wireless networks and sending data to a cloud platform [19, 20]. The IOT medical technology would inform not only physicians but patients of real-time data to identify and early prevent issues [20–23]. Nowadays, many ventilatory devices are embedded with electronics, software, sensors, and network connectivity, which collect, provide and exchange information about compliance, ventilatory parameters, and physiological indices. With more reliable tele-monitoring and transmission technology, IOT-based management of continuous positive airway pressure (CPAP) in obstructive sleep apnea syndrome (OSA) patients, such as follow-up and tele-monitoring on adherence with the use of IOT, is emerging and yielding some positive findings [24–26]. Duiverman et al. [27] conducted the first RCT about home initiation with the help of IOT of chronic NIPPV in COPD patients recently, which demonstrated that home titration was safe and non-inferior to in-hospital titration, and reduced costs by over 50% [27].

However, clear conclusions of IOT-based home NIPPV management in COPD patients with CHRF were lacking. The primary aim of this study was to evaluate the effectiveness (in terms of Health-related Quality of Life and compliance) and safety of the IOT-based management of NIPPV for the COPD patients with CHRF. The secondary aim was to determine whether IOT-based NIPPV is more cost-effective and non-inferior to standard management of NIPPV for decreasing PaCO2, risk of mortality, and all-cause hospital readmission.

## Methods

### Study design and patients

This trial was a multicenter, prospective, parallel-group, randomized controlled study recruiting 200 hypercapnia COPD patients with a 1:1 allocation to NIPPV alone or NIPPV plus IOT-based management. The protocol was written in compliance with the Standard Protocol Items: Recommendations for Interventional Trials guidelines (SPIRIT) and the complete SPIRIT checklist for the study is provided in Additional file 1.

The trial was approved by the Medical Ethics Committee of Zhongshan Hospital Fudan University, patients gave informed consent to participate in the study (Additional file 2) and the trial was registered on 17 November 2018 at the Chinese Clinical Trials Registry (ChiCTR1800019536). Patients were recruited from Zhongshan Hospital Fudan University, QingPu Branch of Zhongshan Hospital Affiliated to Fudan University, Xuhui District Central Hospital, The Central Hospital of Min-Hang District, Jiading District Central Hospital, Shanghai Pudong Hospital, Tongren Hospital Affiliated to Shanghai Jiaotong University, Liqun Hospital of Putuo District and followed up for 12 months. The approval of the protocol by other medical ethics committee at each participating center was obtained before recruitment was initiated. Patients were identified for eligibility when admitted to respiratory wards with an acute exacerbation of COPD and decompensated hypercapnic respiratory failure after resolution of the exacerbation and clinical stabilization.

The patients who meet all of the following inclusion criteria were considered eligible: (1) aged 40 to 80 years old; (2) clearly diagnosed patients with severe or very severe COPD (Global Initiative for Chronic Obstructive Lung Disease stage III or IV [1]): Forced Expiratory Volume in 1 s (FEV1)/forced vital capacity (FVC) < 70% and FEV1% < 50% predicted value after 400 mcg short-acting beta2-agonist (salbutamol); (3) combined with chronic respiratory failure (PaCO2 > 50 mmHg) during daytime in the steady state of COPD.

Patients were excluded if they (1) had unstable cardiac hemodynamics, such as acute left heart failure, unstable angina, and cardiogenic shock; (2) combined with typical pulmonary fibrosis, airway tumors, tuberculosis sequelae (lung damage), and other lung diseases; (3) combined with neuromuscular or chest wall disease.

### Randomization and masking

The randomization of the trial was completed at an independent data center (College of Public Health, Fudan University) using a computer-generated random number sequence with an allocation ratio of 1:1 for each group. All investigators at each of the participating hospitals contacted an independent statistician to obtain an identification code and a random number unique to this patient who fulfills the inclusion criteria. IOT-based management of NIPPV couldn’t be blinded, and an effective sham measure was not available. Supervising clinicians who performed NIPPV and IOT-based management were aware of the treatment assignment of every participant. The staff members conducting the outcome assessments and statistical analysts were blinded to treatment assignment.

### Intervention

In both groups, oxygen therapy (Airsep Oxygen Concentrator VisionAire 5L) was entrained into the NIPPV circuit and oxygen flow rate was initiated to control hypoxemia and hypoventilation aiming to maintain SpO2 > 88%. All patients were instructed to use oxygen therapy for at least 12 h daily. NIPPV was initiated by bi-level positive airway pressure (BiPAP) in the spontaneous/timed mode (Curative Lotus ST30) and adjusted the settings with the aim to: (1) maximally support respiration; (2) reduce at least 10 mmHg PaCO2 compared with the first night of spontaneous breathing or achieve normocapnia. We gradually increased the IPAP during trials to a maximal tolerated level aiming to tidal volume 8 ml/kg and to the maximal level that is tolerated by the patient. Expiratory positive airway pressure (EPAP) was started at 5 cmH2O. The respiratory rate was set as close as possible to the RR of the patient and the inspiration to expiration time was 1:3. All patients were initially given ventilation by means of a nasal, oral-nasal, or full-face mask according to patient preference and humidification to maximize comfort. They were advised to use NIPPV intermittently for at least 8 h per day, preferably continuous use during sleep, but usage during daytime was also accepted. The patient was discharged home once the gas exchange goals were achieved.

Patients in the control group received usual NIPPV management. Patients or their care providers underwent NIPPV training on NIPPV application and operation to enable support for home use of NIPPV prior to discharge. This included information on how to put on the mask, how to turn the device on and off, and the correct cleaning of the tubes, masks, and humidifier. When necessary, ventilator providers joined the patients at home to install the ventilator. Moreover, patients could contact the doctor by 24-h helpline in case of technical problems with the ventilator, or oxygen treatment.

**Fig. 1:**
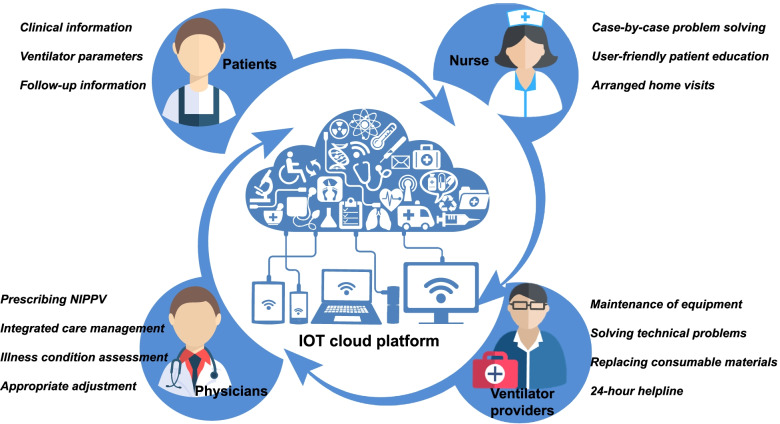
IOT-based management of NIPPV

**Fig. 2:**
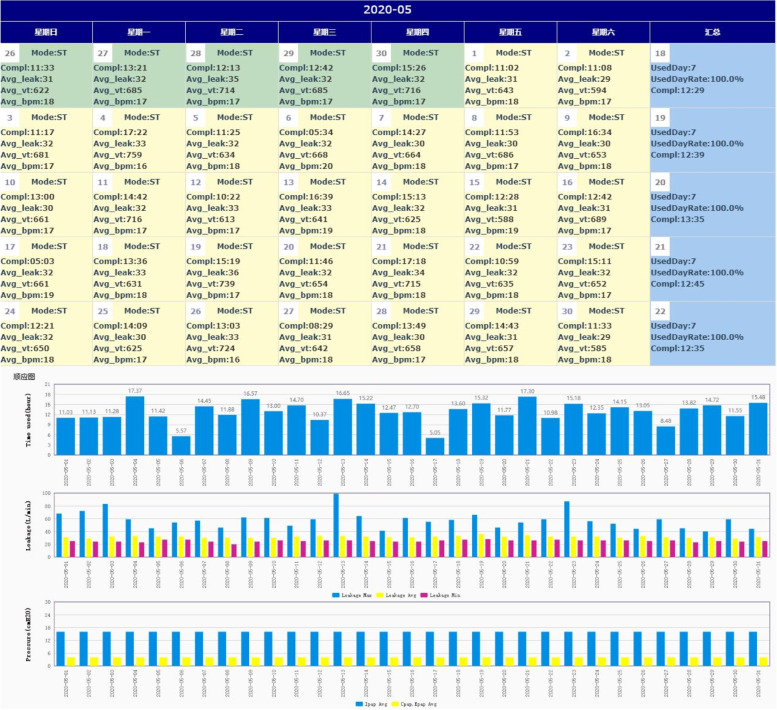
The IOT cloud platform. BPM, breaths per minute; VT, tidal volume

In the intervention group, the optimized titration protocol of ventilator parameters in the hospital was the same as the control group. But patients would receive IOT-based management NIPPV therapy (Fig. 1). Medical management teams would establish a real-time monitoring IOT cloud platform (Suzhou Curative Medical Technology Limited Corporation) including clinical information, ventilator parameters, daily report, and follow-up information. Daily information was retrieved remotely via the 4th generation mobile communication technology (4G) system clicked on the back of the ventilator, which were sent to the secured platform including data collector, parse server, automatic processing server, data storage center, application interface server, and web server. This assessment of daily individual risk was associated with IOT tele-monitoring providing basic usage time, NIPPV pressures, mask leak, breaths per minute (BPM), tidal volume (VT), and residual respiratory events (Fig. 2). The IOT cloud platform allocated clinical information and daily report to medical staff members based on automatic processing of device data. The suggestions of risk intervention were also provided in the event of side effects, leaks, and lack of efficacy. Physicians were in charge of the complete integrated care management and appropriate adjustment of NIPPV treatment based on platform information and communication of patients. After the assessment of physicians, a specialized nurse practitioner would contact the patient, providing case-by-case problem-solving and specific interventions about how to minimize side effects and to improve compliance (interfaces changing, the addition of humidifiers, and so on). Arranged home visits were organized at 1, 4, and 8 months after the NIPPV was initiated, and patients were contacted by telephone every month. The nurse practitioner would provide user-friendly education programs, monitor health status, and ensure adherence to therapy at all home visits. Additionally, ventilator providers would detect problems with technical devices and do re-assessments of technical control of the equipment at all follow-up visits. Moreover, ventilator providers would replace consumable materials and provide 24-h helpline in case of technical problems with the ventilator or long-term oxygen treatment.

General therapy for COPD, such as smoking cessation and pharmacologic therapy will be standardized according to the national treatment guidelines [28] in both groups during the study period. Pharmacological therapy will be guided by symptoms, risk of exacerbations, side effects, comorbidities, and the patient’s response.

### Follow-up and endpoints

**Fig. 3:**
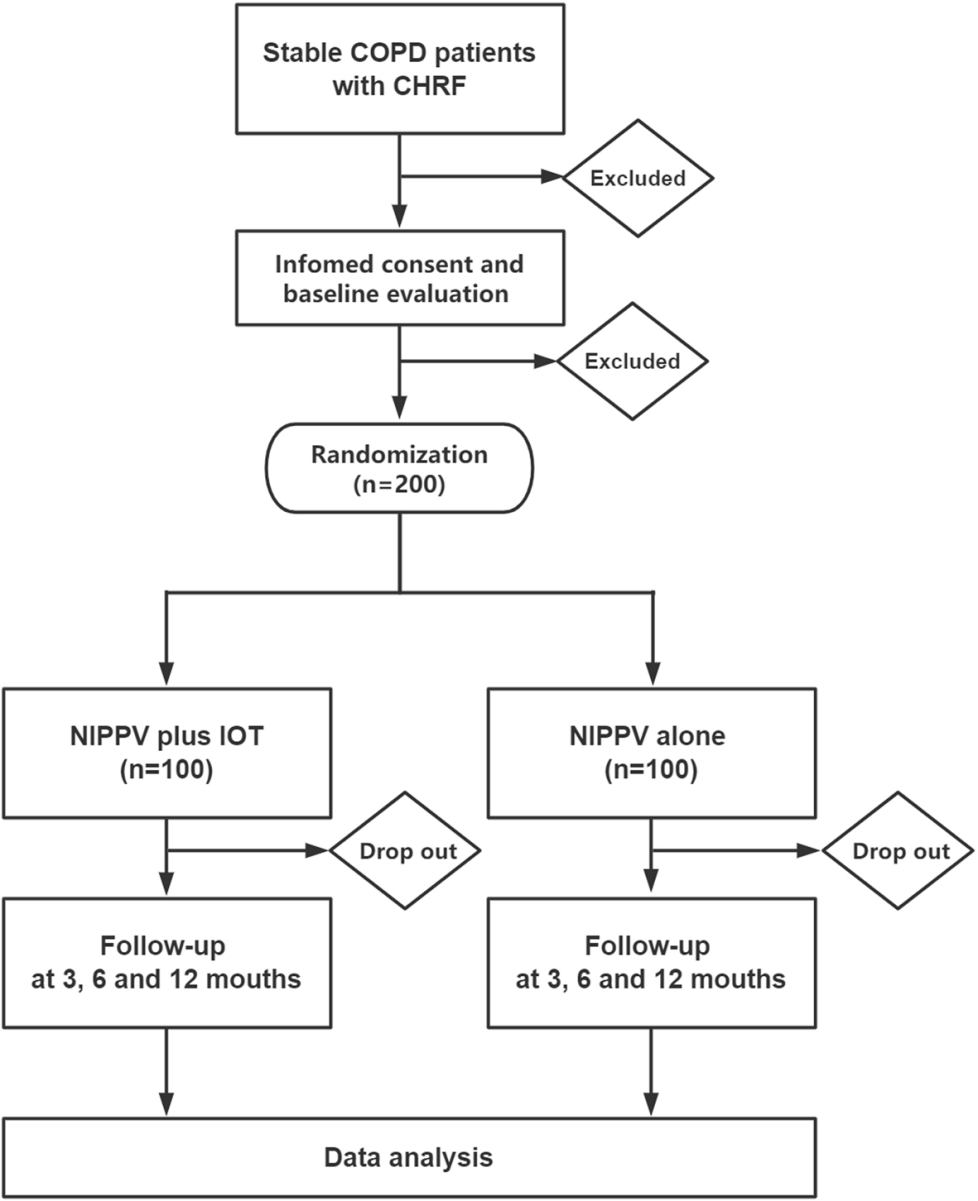
The study flow diagram. NIPPV, noninvasive positive pressure ventilation; COPD, chronic obstructive pulmonary disease; IOT, Internet of things; CHRF, chronic hypercapnic respiratory failure

The SPIRIT schedule for this trial was given in Table 1 and the study flow diagram was shown in Fig. 3. All eligible patients enrolled in the study underwent a baseline assessment of demographics, previous history, health-related quality of life, and measurement of chest CT, lung function, electrocardiogram, echocardiography, routine laboratory tests, and daytime gas exchange. All patients from both groups were admitted to the hospital for the follow-up visits to assess the measurements and ensure optimized medical treatment, including NIPPV. Regular follow-up visits were scheduled at 3, 6, and 12 months after the NIPPV was initiated. We provided a convenient follow-up process by setting a specialty advance appointment clinic.

**Table 1:** SPIRIT schedule of enrollment, intervention, and assessments

Timepoint	Study period					
	**Enrolment**	**Allocation**	**Post-allocation**			**Close-out**
	**Day 0**	**Day 1**	***3 months***	***6 months***	***12 months***	***12 months***
**Enrolment:**						
** Eligibility screen**	X					
** Informed consent**	X					
** Allocation**		X				
**Interventions:**						
** NIPPV plus IOT**		X	X	X	X	
** NIPPV alone**		X	X	X	X	
**ASSESSMENTS:**						
** Demographics**	X					
** Comorbidity**	X					
** Vital signs**		X	X	X	X	
** Hospital readmission**		X	X	X	X	
** Exacerbation**		X	X	X	X	
** Survival status**		X	X	X	X	
** Arterial blood gases**		X	X	X	X	
** Lung function**		X			X	
** SRI score**		X	X	X	X	
** CAT score**		X	X	X	X	
** mMRC score**		X	X	X	X	
** cNAT score**		X	X	X	X	
** Compliance**		X	X	X	X	
** Chest CT**		X			X	
** Electrocardiogram**		X			X	
** Echocardiography**		X			X	
** Routine laboratory tests**		X			X	
** Cost-effectiveness**		X	X	X	X	
** Safety**		X	X	X	X	

*NIPPV* noninvasive positive pressure ventilation, *IOT* Internet of things, *SRI* severe respiratory insufficiency questionnaire, *MRC* Medical Research Council score, *CAT* COPD assessment test, *cNAT* COPD nocturnal symptom assessment test

The enrolled patients who are lost to follow-up, suffered serious adverse events, withdraw consent, or met endpoints will be regarded as a withdrawal. The Intervention and follow-up was terminated If one of the endpoints occurs: (1) death; (2) did not receive intervention as randomized; (3) the need for endotracheal intubation and invasive ventilation during exacerbation; (4) pneumothorax; (5) had active unstable coronary artery syndrome or cerebrovascular disease; (6) unable to tolerate noninvasive ventilation due to surgery and so on; and (7) cognitive impairment or unstable psychiatric morbidity.

### Outcome and assessments

The primary study outcome was the SRI questionnaire, a 49-item questionnaire specifically designed for chronic respiratory failure patients with home mechanical ventilation. The domain scores are calculated by transforming the mean item score into a percentage ranging from 0 (worst quality of life) to 100 (best quality of life) [29, 30]. SRI questionnaire could not be an outcome but the score of SRI questionnaire.

Secondary outcomes were compliance with the ventilator, change in arterial PaCO2 and PaO2, change in lung function, time to readmission or death within 12 months from any cause, all-cause mortality, exacerbation and hospitalization frequency, safety analysis, cost-effectiveness analysis, and other health-related quality of life, measured by the modified Medical Research Council score (MRC) [31] to assess dyspnea (0 = no dyspnea, 4 = dyspnea at rest), the COPD assessment test (CAT) [32, 33], and COPD nocturnal symptom assessment test (cNAT).

Adherence with the ventilator was observed by monitoring the daily usage, usage days, ventilator settings, and other parameters in the IOT platform. Arterial blood gas analysis was taken during the daytime at rest without oxygen or ventilatory support unless patients were unable to stop ventilatory support even for short periods. Lung function was assessed according to national guidelines [28], including FEV1, FEV1% predicted, FVC, FVC % predicted, FEV1/FVC, total lung capacity, and residual volume. Hospital readmission and survival status of study patients were collected by checking medical records from the hospital or the general practitioner at each follow-up visit. For patients lost to clinical follow-up, each study center collected data from the national population register.

Occurrence time, severity, duration, adopted measure, and outcome of the adverse event occurring during the study should be recorded in the adverse events form according to the actual circumstances (Additional file 2). The common adverse events included facial rash, nasal ulceration, dry eyes, conjunctivitis, nasal stuffiness, nosebleed, gastric distension and device/mask intolerance [11, 17]. Serious adverse events were life-threatening, result in death, persistent, and significant disability, or incapacity, or make the participants’ hospitalization.

The main end-point in the cost-effectiveness analysis was cost per admission avoided. The direct and indirect costs included (1) healthcare costs for all hospital readmission; (2) healthcare costs for NIPPV-related medical equipment, such as the ventilatory; (3) healthcare costs for IOT medical equipment, such as tele-monitoring equipment and IOT platform; and (4)healthcare costs for IOT management, such as caregiver the time spent with the patient to initiate, titrate and adapt the NIPPV during the set-up and during the follow-up, maintenance, and support costs.

### Data management and quality control

At each study center, researchers added patient information to a dedicated pseudonymous paper case report form promptly and synchronously with input into the electronic case report form stored in locked file cabinets in areas with limited access. A coordinating and data monitoring committee has been established to record the occurrence of unexpected problems and to store, monitor, and manage the integrity, security, and authenticity of data. All members in the committee were independent of the study funders and declared no competing interests. The committee made safety and progress reports every week. Protocol amendments and the interim analyses would be decided based on the consultation.

### Sample size calculation and statistical analysis

The sample size was based on previous trials [7, 9, 10], assuming that SRI mean score was about 50 in usual NIPPV care after 1 year of treatment. We defined a non-inferiority margin of 5 for the difference in SRI mean score between the two groups, as the difference was found in previous researches about the effect of NIPPV with oxygen therapy vs oxygen therapy alone [9, 10]. On the assumption of a Standard Deviation (SD) of 20 [27] and a loss to follow-up of 25%, a sample size of 100 participants per group was needed to detect a difference of at least 5 between groups with a one-sided alpha of 0.025 and a beta of 0.1.

Continuous variables will be presented as mean and SD for normally distributed data or median and interquartile range (IQR) for nonnormally distributed data. Categorical variables will be presented as absolute numbers and percentages. Differences in continuous variables between two groups will be tested with the Student *t* tests or Mann–Whitney *U* test as appropriate.

The categorical variables will be compared by chi-square tests or Fisher’s exact tests as appropriate. We will use a general linear repeated measures analysis of variance with a Bonferroni correction or a paired t-test to analyze assessments changes within a group over time. The primary and appropriate secondary outcome analyses, such as compliance with the ventilator, change in arterial PaCO2 and lung function, will be performed including all randomized patients according to the intention-to-treat (ITT) principle. Missing data will be handled using the “last observation carried forward” method. We will use a linear mixed model to analyze the mean difference between the groups. When the conditions for use of the linear mixed model are not appropriate, a generalized estimating equation with correction for the baseline value and minimization variables (age, body mass index, frequency of COPD readmissions over 12 months) will be used. Time from randomization to death will be performed according to the ITT principle using the Kaplan–Meier approach and the log rank test. Hazard rate will be analyzed using a Cox proportional hazards regression model adjusted for minimization covariance. When the conditions for use of are not appropriate, the time-dependent cox regression model will be used. Safety analyses will be performed on all randomized patients. Additionally, per-protocol analyses of all outcomes will be analyzed as a sensitivity analysis, including patients finishing the study according to the protocol.

Two-sided P values less than 0.05 will be considered to be statistically significant. Statistical analyses will be performed using SPSS software (version 25.0, IBM SPSS).

## Discussion

The need to increase efficiency, improve the quality of life and to reduce healthcare costs has prompted the development of IOT-based home mechanical ventilation. IOT-based management of NIPPV should be available, ensuring safety, feasibility, and effectiveness to face different patients’ needs. Much more research is needed before considering IOT a real improvement.

Recently, some studies about OSA, neuromuscular or thoracic cage disease have yielded positive findings. Pinto et al. [34] showed that IOT-based tele-monitoring of compliance and ventilator parameters in amyotrophic lateral sclerosis patients offered probable favorable implications on costs, survival, and functional status, but not compliance. Furthermore, a prospective trial [25] about OSA demonstrated that tele-monitoring of CPAP uses with automated feedback messaging improved 90-day compliance in OSA patients. Pépin et al. [24] also found that a significant increase of CPAP adherence and patient-centered outcomes in favor of the multimodal tele-monitoring in OSA patients with high cardiovascular risk. Nevertheless, clear conclusions based on randomized controlled trials of IOT-based management in COPD patients with NIPPV at home are only a few. Generally, the physicians in those studies usually visit patient to solve problems as necessary [24, 26], contact through telephone or just send a message to the patient to improve use [25] in case of dissatisfactory usage or automatic alarm.

In this study, we would establish a real-time monitoring IOT cloud platform including clinical information, ventilator parameters, daily report and follow-up information. Our management teams including physicians, nurse practitioners and ventilator providers would together provide complete integrated care management based IOT cloud platform. It would be more available to face different patients’ needs and would enhance communication between patients, physicians, nurse practitioners and ventilator providers.

This study protocol has limitations which must be addressed. First, blinding of investigators was not possible due to IOT-based management of NIPPV, and it could induce bias. Nevertheless, the staff members conducting the outcome assessments would be blinded to the intervention. Moreover, all data analyses would be performed in a blinded fashion. Second, between-physicians differences in the experience of NIPPV management might affect the results of this study. IOT cloud platform would provide available information and enhance communication between patients, physicians, nurse practitioners and ventilator providers. However, the integrated care managements did not represent a criterion, but provided a personalized care. Third, appropriate adjustment of NIPPV treatment need home visit or hospitalization. Remotely adjusting of ventilator parameters was not available and needs more research. Fourth, several confounding factors associated with general therapy for stable COPD, such as pharmacologic therapy and pulmonary rehabilitation, were only suggested and not protocolized. The general therapy was to be performed in accordance with routine clinical care and pharmacologic therapy was recorded at each follow-up.

In conclusion, this study will be the first and largest randomized trial done in China to evaluate the effectiveness and safety of the IOT-based management of NIPPV for COPD patients with chronic respiratory failure. The results will improve home NIPPV treatment and management in the future.

## Trial status

Currently, participant recruitment is ongoing. Recruitment began in January 2019. The recruitment will be completed in January 2022. The results will be published as soon as possible after the analysis is completed. This protocol version number is Ver.3.

## Supplementary Information

**Additional file 1.** (DOC 126 kb)

**Additional file 2.** (DOCX 76 kb)

## Data Availability

During the study, the data and materials are available from the corresponding author on reasonable request. After the study, the results of this trial will be published in peer-reviewed journals and presented at national and/or international conferences.

## Abbreviations

NIPPV: Noninvasive positive pressure ventilation
COPD: Chronic obstructive pulmonary disease
IOT: Internet of things
SRI: Severe respiratory insufficiency questionnaire
CHRF: Chronic hypercapnic respiratory failure
IPAP: Inspiratory positive airway pressure
CPAP: Continuous positive airway pressure
OSA: Sleep apnea syndrome
FEV1: Forced expiratory volume in 1 s
FVC: Forced vital capacity
BiPAP: Bi-level positive airway pressure
EPAP: Expiratory positive airway pressure
4G: 4Th generation mobile communication technology
BPM: Breaths per minute
VT: Tidal volume
MRC: Medical Research Council score
CAT: COPD assessment test
cNAT: COPD nocturnal symptom assessment test
SD: Standard deviation
IQR: Interquartile range
ITT: Intention-to-treat

## References

[1] Vogelmeier CF, Criner GJ, Martinez FJ, Anzueto A, Barnes PJ, Bourbeau J (2017). Global Strategy for the Diagnosis, Management, and Prevention of Chronic Obstructive Lung Disease 2017 Report. GOLD Executive Summary. Am J Respir Crit Care Med.

[2] Struik FM, Lacasse Y, Goldstein R, Kerstjens HM, Wijkstra PJ. Nocturnal non-invasive positive pressure ventilation for stable chronic obstructive pulmonary disease. Cochrane Database Syst Rev. 2013(6):Cd002878. 10.1002/14651858.CD002878.pub2. 10.1002/14651858.CD002878.pub2 PMC6999800 23766138

[3] Casanova C, Celli BR, Tost L, Soriano E, Abreu J, Velasco V (2000). Long-term controlled trial of nocturnal nasal positive pressure ventilation in patients with severe COPD. Chest.

[4] Clini E, Sturani C, Rossi A, Viaggi S, Corrado A, Donner CF (2002). The Italian multicentre study on noninvasive ventilation in chronic obstructive pulmonary disease patients. Eur Respir J.

[5] Duiverman ML, Wempe JB, Bladder G, Jansen DF, Kerstjens HA, Zijlstra JG (2008). Nocturnal non-invasive ventilation in addition to rehabilitation in hypercapnic patients with COPD. Thorax.

[6] McEvoy RD, Pierce RJ, Hillman D, Esterman A, Ellis EE, Catcheside PG (2009). Nocturnal non-invasive nasal ventilation in stable hypercapnic COPD: a randomised controlled trial. Thorax.

[7] Struik FM, Sprooten RT, Kerstjens HA, Bladder G, Zijnen M, Asin J (2014). Nocturnal non-invasive ventilation in COPD patients with prolonged hypercapnia after ventilatory support for acute respiratory failure: a randomised, controlled, parallel-group study. Thorax.

[8] Sin DD, Wong E, Mayers I, Lien DC, Feeny D, Cheung H (2007). Effects of nocturnal noninvasive mechanical ventilation on heart rate variability of patients with advanced COPD. Chest.

[9] Murphy PB, Rehal S, Arbane G, Bourke S, Calverley PMA, Crook AM (2017). Effect of home noninvasive ventilation with oxygen therapy vs oxygen therapy alone on hospital readmission or death after an acute COPD exacerbation: a randomized clinical trial. JAMA.

[10] Köhnlein T, Windisch W, Köhler D, Drabik A, Geiseler J, Hartl S (2014). Non-invasive positive pressure ventilation for the treatment of severe stable chronic obstructive pulmonary disease: a prospective, multicentre, randomised, controlled clinical trial. Lancet Respir Med.

[11] Wilson ME, Dobler CC, Morrow AS, Beuschel B, Alsawas M, Benkhadra R (2020). Association of home noninvasive positive pressure ventilation with clinical outcomes in chronic obstructive pulmonary disease: a systematic review and meta-analysis. JAMA.

[12] Ergan B, Oczkowski S, Rochwerg B, Carlucci A, Chatwin M, Clini E (2019). European Respiratory Society guidelines on long-term home non-invasive ventilation for management of COPD. Eur Respir J.

[13] Macrea M, Oczkowski S, Rochwerg B, Branson RD, Celli B, Coleman  JM (2020). Long-term noninvasive ventilation in chronic stable hypercapnic chronic obstructive pulmonary disease. An Official American Thoracic Society Clinical Practice Guideline. Am J Respir Crit Care Med.

[14] Raveling T, Vonk J, Struik FM, Goldstein R, Kerstjens HA, Wijkstra PJ (2021). Chronic non-invasive ventilation for chronic obstructive pulmonary disease. Cochrane Database Syst Rev.

[15] Farre R, Lloyd-Owen SJ, Ambrosino N, Donaldson G, Escarrabill J, Fauroux B (2005). Quality control of equipment in home mechanical ventilation: a European survey. Eur Respir J.

[16] Ambrosino N, Vitacca M, Dreher M, Isetta V, Montserrat JM, Tonia T (2016). Tele-monitoring of ventilator-dependent patients: a European Respiratory Society Statement. Eur Respir J.

[17] Cheng S-L, Chan VL, Chu C-M (2012). Compliance with home non-invasive ventilation. Respirology (Carlton, Vic).

[18] Borel JC, Pelletier J, Taleux N, Briault A, Arnol N, Pison C (2015). Parameters recorded by software of non-invasive ventilators predict COPD exacerbation: a proof-of-concept study. Thorax.

[19] Dimitrov DV (2016). Medical Internet of Things and Big Data in Healthcare. Healthc Inform Res.

[20] Dwivedi AD, Srivastava G, Dhar S, Singh R (2019). A Decentralized Privacy-Preserving Healthcare Blockchain for IoT. Sensors (Basel, Switzerland).

[21] Dorsey ER, Topol EJ (2020). Telemedicine 2020 and the next decade. Lancet.

[22] Jagadeeswari V, Subramaniyaswamy V, Logesh R, Vijayakumar V (2018). A study on medical Internet of Things and Big Data in personalized healthcare system. Health Inf Sci Syst.

[23] Ling Y, An T, Yap LW, Zhu B, Gong S, Cheng W (2020). Disruptive, Soft, Wearable Sensors. Adv Mater Weinheim.

[24] Pépin JL, Jullian-Desayes I, Sapène M, Treptow E, Joyeux-Faure M, Benmerad M (2019). Multimodal Remote Monitoring of High Cardiovascular Risk Patients With OSA Initiating CPAP: A Randomized Trial. Chest.

[25] Hwang D, Chang JW, Benjafield AV, Crocker ME, Kelly C, Becker KA (2018). Effect of Telemedicine Education and Telemonitoring on Continuous Positive Airway Pressure Adherence. The Tele-OSA Randomized Trial. Am J Respir Crit Care Med.

[26] Turino C, de Batlle J, Woehrle H, Mayoral A, Castro-Grattoni AL, Gómez S (2017). Management of continuous positive airway pressure treatment compliance using telemonitoring in obstructive sleep apnoea. Eur Respir J.

[27] Duiverman ML, Vonk JM, Bladder G, van Melle JP, Nieuwenhuis J, Hazenberg A, et al. Home initiation of chronic non-invasive ventilation in COPD patients with chronic hypercapnic respiratory failure: a randomised controlled trial. Thorax. 2020;75(3):244–52. 10.1136/thoraxjnl-2019-213303 PMC7063397 31484786

[28] From the Global Strategy for the Diagnosis, Management and Prevention of COPD, Global Initiative for Chronic Obstructive Lung Disease (GOLD). 2021. Available: https://goldcopd.org/2021-gold-reports/.

[29] Ghosh D, Rzehak P, Elliott MW, Windisch W (2012). Validation of the English Severe Respiratory Insufficiency Questionnaire. Eur Respir J.

[30] Windisch W, Freidel K, Schucher B, Baumann H, Wiebel M, Matthys H (2003). The Severe Respiratory Insufficiency (SRI) Questionnaire: a specific measure of health-related quality of life in patients receiving home mechanical ventilation. J Clin Epidemiol.

[31] Bestall JC, Paul EA, Garrod R, Garnham R, Jones PW, Wedzicha JA (1999). Usefulness of the Medical Research Council (MRC) dyspnoea scale as a measure of disability in patients with chronic obstructive pulmonary disease. Thorax.

[32] Gupta N, Pinto LM, Morogan A, Bourbeau J (2014). The COPD assessment test: a systematic review. Eur Respir J.

[33] Karloh M, Fleig Mayer A, Maurici R, Pizzichini MMM, Jones PW, Pizzichini E (2016). The COPD Assessment Test: What Do We Know So Far?: A Systematic Review and Meta-Analysis About Clinical Outcomes Prediction and Classification of Patients Into GOLD Stages. Chest.

[34] Pinto A, Almeida JP, Pinto S, Pereira J, Oliveira AG, de Carvalho M (2010). Home telemonitoring of non-invasive ventilation decreases healthcare utilisation in a prospective controlled trial of patients with amyotrophic lateral sclerosis. J Neurol Neurosurg Psychiatry.

