# Supplementary material for: Internet of things-based home noninvasive ventilation in COPD patients with hypercapnic chronic respiratory failure: study protocol for a randomized controlled trial
Source: Trials. 2022 May 12;23:393. doi: 10.1186/s13063-022-06372-z (PMC9097410; doi:10.1186/s13063-022-06372-z)
Supplement: Supplementary file 2 — Additional file 2. (DOCX 76 kb) [file 13063_2022_6372_MOESM2_ESM.docx]

**基于物联网的家庭吸氧联合无创正压通气治疗慢阻肺合并慢性呼吸衰竭的随机对照前瞻性研究**

**Internet of things-based home noninvasive positive pressure ventilation in COPD patients with hypercapnic chronic respiratory failure**

**病例登记表**

**Case Report Form Ver.3.**

**病例登记号**

**□□□□□**

**Study center:**

- 复旦大学附属中山医院 Zhongshan Hospital, Fudan University
- 徐汇中心医院 Xuhui District Central Hospital
- 闵行中心医院 Minhang District Central Hospital
- 嘉定中心医院 Jiading District Central Hospital
- 青浦中心医院 Zhongshan Hospital, Qingpu Branch, Fudan University
- 上海浦东医院 Shanghai Pudong Hospital
- 浦东人民医院 Pudong people's hospital
- 交大同仁医院 Tongren Hospital Affiliated to Shanghai Jiaotong University
- 普陀利群医院 Putuo liqun hospital
- 昆山医院 Kunshan hospital
- 第七人民医院 Shanghai Seventh People's Hospital

**访视 1（基线数据baseline）**

调查日期Date：□□□□年□□月□□日

**审核入排选标准，符合所有入选标准，无排除标准** **□是** **□否**

**Inclusion criteria**

| **是yes** | **否no** | **入选标准Inclusion criteria** |
| --- | --- | --- |
|  |  |  |
| **☐** | **☐** | 年龄 40 岁**～**80 岁，性别不限Aged 40 to 80 years old |
|  |  |  |
| **☐** | **☐** | 确诊断的重度或极重度慢阻肺患者（吸入支气管舒张剂后）： |
|  |  | FEV1/FVC<70%，FEV1%预计值<50% |
|  |  | Clearly diagnosed patients with severe or very severe COPD (Global Initiative for Chronic Obstructive Lung Disease stage III or IV) |
| **☐** | **☐** | II 型呼吸衰竭：动脉血气分析 PaCO2>50mmHg |
|  |  | Combined with chronic respiratory failure (PaCO2> 50mmHg) |
| **是** | **否** | **排除标准Exclusion criteria** |
|  |  |  |
| **☐** | **☐** | 心脏血流动力学不稳定患者，如急性左心衰，不稳定型心绞痛； |
|  |  | had unstable cardiac hemodynamics, such as acute left heart failure, unstable angina, etc |
| **☐** | **☐** | 合并有典型肺纤维化、气道内肿瘤、结核后遗症（毁损肺）等肺病 |
|  |  | 疾病； |
|  |  | combined with typical pulmonary fibrosis, airway tumors, tuberculosis sequelae (lung damage) and other lung diseases |
| **☐** | **☐** | 患有神经肌肉疾病及严重脑血管意外后遗症者。 |
|  |  | combined with neuromuscular diseases and severe cerebrovascular accident sequelae |

**分组group**

☐ 吸氧+无创机械通气组NIPPV alone

- 吸氧+无创机械通气+物联网+家居医疗服组 NIPPV plus IOT-based management

一、一般情况Demographics

姓名缩写name：___________

性别gender： 1□ 男Male 2□ 女Female 民族Nationality：_________

出生日期Birthdate：□□□□年□□月□□日

身高Height：□□□厘米 体重weight：□□□.□公斤

心率Heart Rate □□□次/分 血压Blood pressure □□□/□□□mmHg

二、吸烟史smoking history

□ 有 ___包/日___年，已戒___年 □ 无

三、职业接触史及个人史Occupation

是否职业接触粉尘或有害气体？□是 □否

居住环境是否接触粉尘或有害气体？ □是 □否

Exposure to dust or harmful gases

过敏史：过敏性鼻炎 □有 □无； 其他□有 □无

allergic history

四、家族史family history

父母兄弟姐妹是否有被诊断下列疾病：

1□慢性支气管炎 2□肺气肿 3□COPD 4□哮喘 5□先天性肺囊肿 6□支气管扩张症 7□睡眠呼吸暂停 8□肺癌 9□不确定 10□均无如是，为何人？

1□Chronic bronchitis 2□ emphysema 3□COPD 4□ asthma 5□ congenital pulmonary cysts 6□ bronchiectasis 7□ sleep apnea 8□ lung cancer

□ 父___ □ 母___ □ 兄弟姐妹___

五、症状评分Quality of Life

SRI 评分 ___________

CAT 评分 ___________

mMRC 评分 ___________

cNAT 评分 ___________

六、心电图Electrocardiograph（日期：_______年______月_______日）

结果_______________________________

□有临床意义； □无临床意义

七、心超echocardiography（日期：_______年______月_______日）

结果_______________________________

肺动脉高压 pulmonary arterial hypertension □有 _________mmHg（估测） □无

1. 动脉血气分析arterial blood gas（日期：_______年______月_______日）

pH __________

PaCO2__________mmHg HCO3- __________ mmol/L

PaO2 __________ mmHg SaO2 __________ %

| 九、肺功能（吸入支气管扩张剂后）  Pulmonary function (after inhalation of bronchodilator) | | |  |  |
| --- | --- | --- | --- | --- |
| 日期： _________年_________月_________日 | | | | 检查号：_____________ |
| FEV1 | ________L | ________%预计值 | FVC | _______L ______%预计值 |
| FEV1/FVC | ________% | ________%预计值 | IC | _______L ______%预计值 |
| VC | ________L | ________%预计值 | FRC | _______L ______%预计值 |
| RV | ________L | ________%预计值 | TLC | _______L ______%预计值 |
| RV/TLC | ________L | ________%预计值 | DLCO | _______L ______%预计值 |
| KCO | ________L | ________%预计值 |  |  |

十、胸部 CT Chest CT（日期：_______年______ 月______ 日 检查号：______）

结果：______________________________________

十一、血化验Blood Routine Test

| 肝功能 | ALT_____u/L | AST u/L | 总胆红TB ______umol/L |
| --- | --- | --- | --- |
|  | 白蛋白albumin g/L | LDH u/L | 直接胆红素DB_____umol/L |
| 肾功能 | 尿BUN mmol/L | creatinine__umol/L | 尿酸UA ______umol/L |
| 电解质 | 钠Na mmol/L | 钾K mmol/L | 氯Cl mmol/L |
|  | 钙Ca mmol/L | 磷P mmol/L | 镁Mg mmol/L |
| 血糖blood glucose mmol/L  总胆固醇TC mmol/L 甘油三脂TG mmol/L | | | |

十二、合并症complication

| 高血压 hypertension | □有 | □无 |
| --- | --- | --- |
| 高脂血症 hyperlipidemia | □有 | □无 |
| 冠心病 coronary heart disease | □有 | □无 |
| 脑卒中 stroke | □有 | □无 |
| 糖尿病 diabetes | □有 | □无 |
| 睡眠呼吸暂停 sleep apnea | □有 | □无 |
| 骨质疏松 osteoporosis | □有 | □无 |
| 其他others | □有 | □无 |

十三、吸氧设置

氧流Oxygen flow ________L/min每日time ________h SpO2 ________%

十四、呼吸机设置Ventilator

EPAP ________cmH2O IPAP ________cmH2O

RR _________次/分 I:E__________

十五、物联网设置 IOT management

□使用 □未使用

**访视 follow-up 1（第 90 天±14 天）**

调查日期date：□□□□年□□月□□日

一、一般情况Demographics

姓名缩写：___________

身高：□□□厘米 体重：□□□.□公斤

心率 □□□次/分 血压 □□□/□□□mmHg

二、吸烟史 smoking history

吸烟状态有无变化 □有 □无

三、 症状评分Quality of Life

SRI 评分 ___________

CAT 评分 ___________

mMRC 评分 ___________

cNAT 评分 ______________

四、动脉血气分析arterial blood gas（日期：________年________月________日）

pH __________

PaCO2__________mmHg HCO3^-^__________mmol/L

PaO2 __________ mmHg SaO2 __________%

五、肺功能Pulmonary function

日期：_______ 年_______月_______日 检查号：______

| FEV1 ______L______%预计值 FVC ______L_______ %预计值 |
| --- |
| FEV1/FVC ______%______%预计值 IC ______L_______ %预计值 |
| VC ______%______%预计值 FRC ______L_______ %预计值 |

| RV ______L______%预计值 TLC ______L_______ %预计值 |
| --- |
| RV/TLC ______%______%预计值 DLCO ______L_______ %预计值 |
| KCO ______%______%预计值 |

六、吸氧设置oxygen

| □ 有变化 | 氧流量________L/min | 每日 ________h |
| --- | --- | --- |
| □ 无变化 | | |

1. 呼吸机设置Ventilator

| □ 有变化 | EPAP_______cmH2O | IPAP _____cmH2O  RR _____次/分 | I:E _______ |
| --- | --- | --- | --- |
| □ 无变化 | | | |

八、慢阻肺急性加重AECOPD

□ 有 （□普通住院hospitalization □ICU 住院 □急诊 □门诊）

若住院：住院次数______，累计天数______，累计费用_______

□ 无

九、终点指标Endpoint criteria

□ 有 _______________

□ 无

十、物联网设置 IOT management

□使用 □未使用


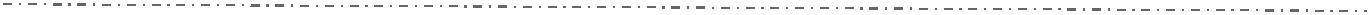


**Follow-up 2（第 180 天±14 天）**

调查日期date：□□□□年□□月□□日

一、一般情况Demographics

姓名缩写：___________

身高：□□□厘米 体重：□□□.□公斤心率 □□□次/分 血压 □□□/□□□mmHg

二、吸烟史smoking history

吸烟状态有无变化 □有 □无

三、 症状评分Quality of Life

SRI 评分 ___________

CAT 评分 ___________

mMRC 评分 ___________

cNAT 评分 ______________

四、动脉血气分析arterial blood gas（日期：______年______月________日）

pH __________

PaCO2__________mmHg HCO3^-^__________mmHg

PaO2 __________ mmHg SaO2 __________%

五、肺功能Pulmonary function

日期：________ 年________ 月________ 日 检查号：________________

FEV1 ________L ________%预计值 FVC ______L ______%预计值

FEV1/FVC ________% ________%预计值 IC ______L ______%预计值

VC ________L________%预计值 FRC ______L______%预计值

RV ________L________%预计值 TLC ______L______%预计值


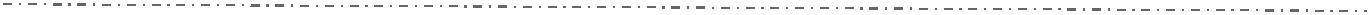


RV/TLC ________L_______%预计值 DLCO______L______%预计值

KCO ________L________%预计值

六、吸氧设置oxygen

□ 有变化 氧流量________L/min 每日 ________h

□ 无变化

七、呼吸机设置Ventilator

EPAP ______cmH2O IPAP _____cmH2O

□ 有变化

RR _____次/分 I:E _______

□ 无变化

八、慢阻肺急性加重 AECOPD

□ 有 （□普通住院 □ICU 住院 □急诊 □门诊）

若住院：住院次数______，累计天数______，累计费用_______

□ 无

九、终点指标Endpoint criteria

□ 有 _______________

□ 无

1. 物联网设置 IOT management

□使用 □未使用

**Follow-up 3（第 360 天±14 天）**

调查日期date：□□□□年□□月□□日

一、一般情况Demographics

姓名缩写：___________

身高：□□□厘米 体重：□□□.□公斤

心率 □□□次/分 血压 □□□/□□□mmHg

二、吸烟史smoking history

吸烟状态有无变化 □有 □无

三、 症状评分Quality of Life

SRI 评分 ______________

CAT 评分 ______________

mMRC 评分 ______________

cNAT 评分 ______________

6 分钟步行距离 ___________ 测试后 Borg 评分 ___________

四、动脉血气分析Quality of Life（日期：______年______月______日）

pH __________

PaCO2__________mmHg HCO3^-^__________mmol/L

PaO2 __________ mmHg SaO2 __________%

1. 心电图Electrocardiogram（日期： ______ 年 ______ 月______ 日）

结果____________

□有临床意义； □无临床意义

六、心超echocardiography（日期： ______ 年 ______ 月______ 日）

结果_______________________________

肺功能脉高压 □有 ________mmHg（估测）□无

七、肺功能lung function

日期：_______ 年_______月_______日 检查号：________________

| FEV1 | ______L ______%预计值 | FVC | ______L | _______ %预计值 |
| --- | --- | --- | --- | --- |
| FEV1/FVC | ______% ______%预计值 | IC | ______L | _______ %预计值 |
| VC | ________L ______%预计值 | FRC | ______L | _______ %预计值 |
| RV | ________L ______%预计值 | TLC | ______L | _______ %预计值 |
| RV/TLC | ________L ______%预计值 | DLCO | ______L | _______ %预计值 |
| KCO | ________L ______%预计值 |  |  |  |

八、胸部 CT Chest CT（日期：______年____月____日；检查号：___________）

结果：______________________________________

九、血化验Blood Routine Test

| 肝功能 | ALT_____u/L | AST u/L | 总胆红TB ______umol/L |
| --- | --- | --- | --- |
|  | 白蛋白albumin g/L | LDH u/L | 直接胆红素DB_____umol/L |
| 肾功能 | 尿BUN mmol/L | creatinine__umol/L | 尿酸UA ______umol/L |
| 电解质 | 钠Na mmol/L | 钾K mmol/L | 氯Cl mmol/L |
|  | 钙Ca mmol/L | 磷P mmol/L | 镁Mg mmol/L |
| 血糖blood glucose mmol/L  总胆固醇TC mmol/L 甘油三脂TG mmol/L | | | |

十、吸氧设置oxygen

□ 有变化 氧流量________L/min 每日 ________h □ 无变化

十一、呼吸机设置Ventilator

□ 有变化 EPAP ______cmH2O IPAP _____ cmH2O RR _____次/分 I:E _______

□ 无变化

十二、慢阻肺急性加重 AECOPD

□ 有 （□普通住院 □ICU 住院 □急诊 □门诊）

若住院：住院次数______，累计天数______，累计费用_______

□ 无

**严重不良事件报告表（Serious Adverse Event Report Form）**

编号：

| **研究相关资料Research related materials** | | | |
| --- | --- | --- | --- |
| 项目名称project | 基于物联网的家庭吸氧联合无创正压通气治疗慢阻肺合并慢性呼吸衰竭的前瞻性随机对照研究  Internet of things-based home noninvasive positive pressure ventilation in COPD patients with hypercapnic chronic respiratory failure | | |
| 研究器械名称 device | CURATIVE ST-30Ventilator; visionaire 5L oxygenerator; | | |
| 研究器械分类classification | Ⅰ类，Ⅱ类，Ⅲ类，其它 | | |
| 临床研究注册编号Research Registration Number | ChiCTR1800019536 | | |
| 首次报告（日期time： 年 月 日）， 随访报告， 总结报告 | | | |
| **申办单位 research center** | | | |
| 申办单位名称 | 复旦大学附属中山医院 上海市呼吸病研究所  Zhongshan Hospital, Fudan University | | |
| 申办单位地址 address | 上海市徐汇区枫林路180号  180 Fenglin Road, Xuhui District, Shanghai | | |
| 电话 phone |  | 邮箱E-Mail |  |
| **研究单位** | | | |
| 研究机构名称 |  | | |
| 研究机构地址 |  | | |
| 电话 |  | 邮箱 |  |
| **受试者Patients** | | | |
| 姓名拼音首字母缩写 |  | | |
| 受试者随机编码 |  | | |
| 出生日期 | 年 月 日 | | |
| 性别 | 男， 女 | | |
| 体重 | 千克 | | |
| 身高 | 厘米 | | |
| **SAE分类 classification** | | | |
| 住院 / 延长住院时间 / 危及生命 / 永久或严重致残 /死亡/ 其他重要医学事件  Hospitalization / Hospitalisation or prolongation of treatment / life-threatening / Persistent or symptomatic disability or incapacity / death / other important medical events | | | |
| 如果死亡，死亡时间： 年 月 日  If death, time of death | | | |

| **SAE名称及描述 If SAE specify:** | | | | | | | | | | | | |
| --- | --- | --- | --- | --- | --- | --- | --- | --- | --- | --- | --- | --- |
| SAE名称 | | | | | （如可能，请作出诊断，并使用专业术语） | | | | | | | |
| SAE是否预期 | | | | | ¨ 否，¨ 是（已在临床研究方案/知情同意书中说明） | | | | | | | |
| SAE发生时间 Onset Date | | | | | 年 月 日 | | | | | | | |
| SAE获知时间 | | | | | 年 月 日 | | | | | | | |
| SAE描述（包括受试者相关病史，AE的症状/体征、治疗、发生及转归过程/结果和AE可能原因分析，如有更多信息可另附页记录）： | | | | | | | | | | | | |
| **相关实验室/其他检查结果Laboratory/other test results** | | | | | | | | | | | | |
| 实验室/检查项目 | | 结果 | | | | | 单位 | | 检查日期 | | 对结果的说明 | |
|  | |  | | | | |  | |  | |  | |
|  | |  | | | | |  | |  | |  | |
|  | |  | | | | |  | |  | |  | |
| **研究器械使用情况Device usage** | | | | | | | | | | | | |
| 器械名称 | 使用频率 | | | 使用方法 | | | | 首 用  日 期 | | 使用中 | | 停 止  日 期 |
|  |  | | |  | | | | 年 月 日 | | 是/否 | | 年 月 日 |
|  |  | | |  | | | | 年 月 日 | | 是/否 | | 年 月 日 |
| 注1：如为设盲试验，是否紧急破盲：o是，o否à请在“器械名称”栏填写器械编号 | | | | | | | | | | | | |
| 注2：如方案规定需调整研究器械使用频率，请说明： | | | | | | | | | | | | |
| **可能与SAE有关的器械**（如非器械因素导致SAE，此栏内容可不填） | | | | | | | | | | | | |
| 可能与SAE有关的器械名称 | | | | | |  | | | | | | |
| 该器械属于本临床试验的 | | | | | | 研究器械（如果非盲/破盲：试验器械，对照器械），o其他 | | | | | | |
| 该器械的适应证 | | | | | |  | | | | | | |
| 首次使用至SAE发生的时间 | | | | | | 天（如果能够精确计算： 时 分） | | | | | | |
| 末次使用至SAE发生的时间 | | | | | | 天（如果能够精确计算： 时 分） | | | | | | |
| **SAE与研究器械的关系（因果关系）Relationship to Study interventions** | | | | | | | | | | | | |
| 无关 / 可能无关 / 可能有关 / 很可能有关 / 有关 / 现有信息无法判断  Certain/ Unlikely/ Probable / Not related / Possible / Unclassified | | | | | | | | | | | | |
| **采取的措施 Action Taken with Interventions** | | | | | | | | | | | | |
| 无 / 调整研究器械的使用频率 / 暂停研究器械 / 停用研究器械 / 增加新的治疗器械 / 应用非器械治疗 / 延长住院时间 / 修改方案/知情同意书  Intervention unchanged/ Intervention temporarily interrupted/ Frequency increased/ Frequency reduced/ Intervention withdrawn/ Not Known | | | | | | | | | | | | |
| **转归 Outcome** | | | | | | | | | | | | |
| 完全痊愈 / 症状改善 / 症状恶化 / 痊愈 / 有后遗症 / 症状无变化 / 死亡  Recovered/ Recovering with sequelae/ Fatal/ Recovering/ Continuing/ Not Known  尸检： 否 / 是（请附尸检报告） | | | | | | | | | | | | |
| **报告 report** | | | | | | | | | | | | |
| 报告人签字signature | | |  | | | | | | | | | |
| 本次报告日期 | | |  | | | | | | | | | |

知情同意书Consent Form

受试者须知页

Subject notice page

方案名称：基于物联网的家庭吸氧联合无创正压通气治疗慢阻肺合并慢性呼吸衰竭的随机对照前瞻性研究

Project：Internet of things-based home noninvasive positive pressure ventilation in COPD patients with hypercapnic chronic respiratory failure

主要研究者：宋元林

Major investigators: Yuanlin Song

申办者: 复旦大学附属中山医院

Zhongshan Hospital, Fudan University, Shanghai

尊敬的受试者：

Dear subjects:

您被邀请参加 基于物联网的家庭吸氧联合无创正压通气治疗慢阻肺合并慢性呼吸衰竭的随机对照前瞻性研究 研究，该项研究由复旦大学附属中山医院提供支持。请仔细阅读本知情同意书并慎重做出是否参加本项研究的决定。参加这项研究完全是您自主的选择。作为受试者，您必须在加入临床研究前给出您的书面同意书。当您的研究医生或者研究人员和您讨论知情同意书的时候，您可以让他/她给您解释您看不明白的地方。我们鼓励您在做出参与此项研究的决定之前，和您的家人及朋友进行充分讨论。您有权拒绝参加本研究，也可随时退出研究，且不会受到处罚， 也不会失去您应有的权利。若您正在参加别的研究，请告知您的研究医生或者研究人员。本研究的背景、目的、研究过程及其他重要信息如下：

You are invited to participate in the study: Internet of things-based home noninvasive positive pressure ventilation in COPD patients with hypercapnic chronic respiratory failure. The study was supported by Zhongshan Hospital affiliated to Fudan University. Please read this informed consent form carefully and make a careful decision on whether or not to participate in this study. It is your own choice to participate in this study. As a subject, you must give your written consent before joining the clinical study. When your research doctor or researcher discusses the informed consent form with you, you can ask him or her to explain to you what you don't understand. We encourage you to have a full discussion with your family and friends before making the decision to participate in this study. You have the right to refuse to participate in this study, or you can withdraw from the study at any time without being punished or losing your due rights. If you are participating in another study, please inform your research doctor or researcher. The background, purpose, research process and other important information of this study are as follows:

一、 研究背景

Research background

慢性阻塞性肺疾病（慢阻肺）是一种患病率和死亡率较高的疾病，困扰慢阻肺患者的最大问题，是逐年加重的呼吸困难、运动耐量下降、反复的急性加重发作， 进而造成生活质量下降，给患者及家庭带来沉重的负担。

Chronic obstructive pulmonary disease (COPD) is a disease with high morbidity and mortality. the biggest problem troubling patients with COPD is increased dyspnea, decreased exercise tolerance and repeated acute exacerbations year by year, resulting in a decline in the quality of life and a heavy burden on patients and families.

对于重度慢阻肺合并慢性呼吸衰竭患者，长期家庭氧疗能改善其缺氧，纠正 CO2 潴留，提高患者生存率。无创正压通气在治疗慢阻肺患者急性呼吸衰竭方面有较好的作用，可降低患者气管插管率和病死率、减少住院时间。然而，慢阻肺患者多为中老年人群，对于家庭氧疗和呼吸机的使用及调整存在一定困难，影响了依从性及治疗效果。物联网作为一个新兴的信息技术, 借助射频识别、传感器网络、无线数据通信等智能技术, 实现对物体实时、智能化地识别、定位、追踪、监控与管理, 并可触发相应事件。有望指导患者最佳的家庭长期应用无创正压通气和/或氧疗，但目前尚缺乏这方面的研究结果。因此，有必要采用随机前瞻对照的研究探索基于物联

网的家庭无创通气治疗慢阻肺合并呼吸衰竭患者的疗效与安全性。

For patients with severe COPD complicated with chronic respiratory failure, long-term home oxygen therapy can improve their hypoxia, correct CO2 retention and improve the survival rate of patients. Non-invasive positive pressure ventilation plays a good role in the treatment of acute respiratory failure in patients with COPD, which can reduce the rate of endotracheal intubation, mortality and hospital stay. However, most of the patients with COPD are middle-aged and elderly people, and there are some difficulties in the use and adjustment of home oxygen therapy and ventilator, which affect the compliance and therapeutic effect. As an emerging information technology, the Internet of things realizes real-time and intelligent identification, positioning, tracking, monitoring and management of objects with the help of intelligent technologies such as radio frequency identification, sensor networks and wireless data communications, and can trigger corresponding events. It is expected to guide the best family use of non-invasive positive pressure ventilation and / or oxygen therapy for a long time, but there is a lack of research results in this area. Therefore, it is necessary to use a randomized prospective controlled study to explore the efficacy and safety of home non-invasive ventilation based on the Internet of things in the treatment of COPD patients with respiratory failure.

二、 研究目的

Purpose

应用基于物联网的长期家庭吸氧联合无创正压通气治疗合并慢性呼吸衰竭的慢阻肺居家患者进行疾病管理，评价其健康相关的生存质量方面的有效性和安全性。

The primary aim of this study is to evaluate the effectiveness (in terms of Health-related Quality of Life and compliance) and safety of the IOT-based management of NIPPV for the COPD patients with CHRF. The secondary aim is to determine whether IOT-based NIPPV is more cost-effective and non-inferior to standard management of NIPPV for decreasing PaCO2, risk of mortality and all-cause hospital readmission.

三、研究过程

Research process

1. **多少人将参与这项研究？**

**patients**

本研究将在复旦大学附属中山医院本部和青浦分院以及上海多家二级或以上综合医院的住院和门诊开展，预计有 200 人参与，所有患者均由专科医师滴定吸氧浓度和无创正压通气各参数，患者在家庭长期使用。

This study will be carried out in the inpatients and outpatients of Zhongshan Hospital affiliated to Fudan University and Qingpu Branch as well as a number of secondary or above general hospitals in Shanghai. 200 people are expected to participate. All patients were titrated by specialists to titrate the oxygen concentration and the parameters of non-invasive positive pressure ventilation, and the patients were used in the family for a long time.

1. 研究步骤

如果您同意参加本研究，请您签署这份知情同意书。

If you agree to participate in this study, please sign this informed consent form.

整个研究将持续 1 年，您在入组时、入组后第 90 天、180 天和 360 天会进行严重呼吸功能不全问卷（SRI）评分、慢阻肺症状测试（CAT）评分、mMRC 评分、睡眠症状评估（cNAT）、6 分钟步行距离及 Borg 评分、血气分析、心超、心电图、肺功能（可选）、胸部 CT 以及血液体检项目（肝肾功能、电解质、血糖和血脂）等问卷及检查。研究医生会记录您每次随访间期内的急性加重次数、急性加重需要住院次数和天数、急性加重需住 ICU 次数和天数及住院所需费用等信息。

The whole study will last for one year. The severe respiratory insufficiency questionnaire (SRI) score, COPD symptom test (CAT) score, mMRC score, sleep symptom assessment (cNAT), 6-minute walking distance and Borg score, blood gas analysis, echocardiography, electrocardiogram, lung function (optional), chest CT and blood physical examination items (liver and kidney function, electrolytes, blood sugar and blood lipids) were performed on the 90th, 180th and 360th day of your enrollment. The research doctor will record the number of acute exacerbations during each follow-up period, the number and days of hospitalization required for acute exacerbations, the number and days of ICU stay for acute exacerbations, and the cost of hospitalization.

您可以在任何时间选择退出研究而不会失去您本应获得的任何利益。然而，如果在研究途中您决定退出本研究，我们鼓励您先和您的医生商议。如果您出现严重的不良事件，或者您的研究医生觉得继续参加研究不符合您的最佳利益，他/她会决定让您退出研究。申办者或者监管机构也可能在研究期间终止研究。但您的退出不会影响您的正常医疗待遇与权益不受影响。

You can opt out of the study at any time without losing any of the benefits you should have earned. However, if you decide to withdraw from this study during the study, you are encouraged to consult your doctor first. If you have a serious adverse event, or if your research doctor feels that it is not in your best interest to continue to participate in the study, he or she will decide to let you out of the study. Sponsors or regulators may also terminate the study during the study period. However, your withdrawal will not affect your normal medical treatment and rights and interests will not be affected.

如果您因为任何原因从研究中退出，您可能被询问有关您参加研究的情况。如果医生认为需要，您也可能被要求进行实验室检查和体格检查。

If you withdraw from the study for any reason, you may be asked about your participation in the study. If your doctor thinks it is necessary, you may also be required to undergo laboratory and physical examinations.

四、风险与受益

Risk and benefit

1. **参加本研究的风险是什么？**

参加本研究可能给您带来的风险如下。您应该和您的研究医生，或者您愿意， 与您平日照看您的医生讨论一下这些风险。

The risks that may be brought to you by participating in this study are as follows. You should discuss these risks with your research doctor, or if you are willing, with the doctor who usually looks after you.

比如，在研究期间，最初滴定的吸气压及呼气压在家庭使用的过程中，随着病情的变化，操作者的使用技巧等原因，不是患者最适合最能接受的治疗压力，会出

现漏气、口干、鼻面部压伤、气压伤、影响睡眠质量等问题，最终导致治疗失败。解决方法：对每个入组的患者进行随访，对出现的问题进行指导，必要时上门指导其使用，并保证家庭中有人监督治疗的实施。

For example, during the study period, the initial titration of inspiratory pressure and expiratory pressure in the process of family use, with the change of the disease, the operator's skills and other reasons, is not the most suitable for patients to accept the treatment pressure, there will be air leakage, dry mouth, nasal and facial compression injury, air pressure injury, affecting the quality of sleep and other problems, resulting in treatment failure. Solution: follow up each patient in the group, guide the problems, guide their use if necessary, and ensure that someone in the family supervises the implementation of the treatment.

本研究历时 1 年，患者在研究过程中可能会出现多次急性加重，期间痰量增多， 无创正压通气使用可能造成排痰困难，影响患者预后。解决方法：患者每次急性加重均电话与本研究小组联系，本研究小组给予合理建议，必要时上门指导患者及家属。

This study lasted for one year, patients may have many times of acute aggravation in the course of the study, during which the amount of sputum increased, the use of non-invasive positive pressure ventilation may cause sputum discharge difficulties and affect the prognosis of patients. Solution: patients with acute exacerbation each time to contact the research group, the research group to give reasonable advice, if necessary door-to-door guidance to patients and their families.

如果在研究期间您出现任何不适，或病情发生新的变化，或任何意外情况，不管是否与研究有关，均应及时通知您的医生，他/她将对此作出判断并给与适当的医疗处理。如情况紧急，请优先拨打“**120**”送医院急救，并及时通知您的医生，告知您目前的诊疗情况。

If you have any discomfort, new changes in your condition, or any unexpected circumstances during the study, whether related to the study or not, you should inform your doctor in a timely manner, and he / she will make a judgment and give appropriate medical treatment. If the situation is urgent, please give priority to dial "120" to send to the hospital for first aid, and inform your doctor in time to inform you of your current diagnosis and treatment.

您在研究期间需要按时到医院随访，做一些检查，这将会占用您的一些时间， 也可能给您造成麻烦或带来不方便。

During the study, you need to follow up at the hospital on time and do some tests, which will take up some of your time and may cause trouble or inconvenience to you.

1. 参加研究有什么受益？

如果您同意参加本研究，您将有机会获得物联网下动态监测数据报告和研究者的及时提醒推送；所有患者均会接受到专业的治疗方案。同时，我们希望从您参与的本研究中得到的信息在将来能够使您或与您病情相同的病人获益。

If you agree to participate in this study, you will have the opportunity to receive dynamic monitoring data reports under the Internet of things and timely reminders from researchers; all patients will receive professional treatment. At the same time, we hope that the information obtained from your participation in this study will benefit you or patients with the same condition as you in the future.

在研究期间，您将获得为期 1 年免费的制氧机使用权和无创呼吸机使用权。在研究期间，按随访所行的动脉血气分析或指脉氧监测，以及心超、心电图、

肺功能、胸部 CT 平扫、血液体检项目（肝肾功能、电解质、血糖和血脂）等检查均免费。每次随访有 100 元交通补贴，共 4 次。本研究不负担慢阻肺急性加重或研究期间其他疾病导致的相关医疗费用。

During the period of the study, you will receive free access to oxygen machines and non-invasive ventilators for a period of one year. During the study period, arterial blood gas analysis or finger pulse oxygen monitoring, as well as echocardiography, electrocardiogram, pulmonary function, chest CT scan, blood physical examination items (liver and kidney function, electrolytes, blood glucose and blood lipids) were free of charge. There is a transportation subsidy of 100 yuan for each follow-up visit, for a total of 4 times. This study does not bear the related medical expenses caused by acute exacerbation of COPD or other diseases during the study period.

五、研究结果的使用和个人信息的保密

The use of research results and the confidentiality of personal information

在您和其他受试者的理解和协助下，通过本项目研究的结果可能会在医学杂志上发表，但是我们会按照法律的要求为您的研究记录保密。研究受试者的个人信息将受到严格保密，除非应相关法律要求，您个人信息不会被泄露。必要时，政府管理部门和医院伦理委员会及其它相关研究人员可以按规定查阅您的资料。

With the understanding and assistance of you and other subjects, the results of the study through this project may be published in medical journals, but we will keep your research records confidential in accordance with the law. The personal information of the study subjects will be kept strictly confidential and your personal information will not be disclosed unless required by relevant laws. If necessary, the government administration, the hospital ethics committee and other relevant researchers can consult your information in accordance with the regulations.

六、受试者的权利和相关注意事项

Subjects' rights and related matters needing attention

1. **您的权利**

**Rights**

在参加研究的整个过程中，您都是自愿的。如果您决定不参加本研究，也不会

影响您应该得到的其他治疗。如果您决定参加，会要求您在这份书面知情同意书上签字。您有权在试验的任何阶段随时退出试验而不会遭到歧视或受到不公平的待遇， 您相应医疗待遇与权益不受影响。

During the whole process of participating in the study, you volunteered. If you decide not to participate in this study, it will not affect the other treatments you should receive. If you decide to participate, you will be required to sign this written informed consent form. You have the right to withdraw from the trial at any stage of the trial without discrimination or unfair treatment, and your corresponding medical treatment and rights and interests will not be affected.

1. 注意事项

作为受试者，您需要提供有关自身病史和当前身体状况的真实情况；告诉研究医生自己在本次研究期间所发现的任何不适。

As a subject, you need to provide true information about your medical history and current physical condition; tell your research doctor about any discomfort you found during this study.

七、获知信息的相关联系方式

Contact information about information

如果在研究过程中有任何重要的新信息，可能影响您继续参加研究的意愿时， 您的医生将会及时通知您。如果您对自己的研究数据，或研究结束后您希望知道本研究的发现。您可以在任何时间提出有关本项研究的任何问题，并得到相应的解答， 请通过电话 与 医生联系。

If there is any important new information in the course of the study, which may affect your willingness to continue to participate in the study, your doctor will inform you in a timely manner. If you are interested in your own research data, or after the study is over, you would like to know the findings of this study. You can ask any questions about this study at any time and get answers accordingly. Please contact your doctor by phone.

伦理委员会已经审查通过该研究，如果您有与自身权利/权益相关的任何问题， 或者您想反映参与本研究过程中遭遇的困难、不满和忧虑，或者想提供与本研究有关的意见和建议，请联系复旦大学附属中山医院伦理委员会， 联系电话： 021-

64041990 转 3257，电子邮件：[ec@zs-hospital.sh.cn](mailto:ec@zs-hospital.sh.cn)。

The Ethics Committee has reviewed and approved the study. If you have any questions related to your rights / interests, or if you want to reflect the difficulties, grievances and worries encountered in participating in this study, or would like to provide comments and suggestions related to this study, please contact the Ethics Committee of Zhongshan Hospital affiliated to Fudan University, Tel: 021-.

64041990 ext. 3257, email: ec@zs-hospital.sh.cn.

受试者签字页

Subject signature page

知情同意声明：

Informed consent statement:

我已被告知此项研究的目的、背景、过程、风险及获益等情况。我有足够的时间和机会进行提问，问题的答复我很满意。

I have been informed of the purpose, background, process, risks and benefits of the study. I have enough time and opportunity to ask questions, and I am satisfied with the answers.

我也被告知，当我有问题、想反映困难、顾虑、对研究的建议，或想进一步获得信息，或为研究提供帮助时，应当与谁联系。

I was also told who to contact when I have questions, want to reflect difficulties, concerns, suggestions for research, or want to get further information or help with research.

我已经阅读这份知情同意书，并且同意参加本研究。

我知道我可以选择不参加此项研究，或在研究期间的任何时候无需任何理由退出本研究*。*

I have read this informed consent form and agreed to participate in this study.

I know that I can choose not to participate in this study, or withdraw from this study at any time during the study without any reason.

我已知道如果我的状况更差了，或者我出现严重的不良事件，或者我的研究医生觉得继续参加研究不符合我的最佳利益，他/她会决定让我退出研究。无需征得我的同意，资助方或者监管机构也可能在研究期间终止研究。如果发生该情况，医生将及时通知我，研究医生也会与我讨论我的其他选择。

I already know that if my condition gets worse, or if I have a serious adverse event, or if my research doctor feels that it is not in my best interest to continue to participate in the study, he or she will decide to let me out of the study. Funders or regulators may also terminate the study during the study without my consent. If this happens, the doctor will inform me in time, and the research doctor will discuss my other options with me.

我将得到这份知情同意书的副本，上面包含我和研究者的签名。

I will get a copy of this informed consent form, which contains the signatures of me and the researcher.

受试者签名： 日期：

（注：如果受试者无行为能力/限制行为能力时，则需法定代理人签名和签署日期）

Subject signature: date:

(note: if the subject is incapacitated / restricted, the signature and date of signature of the legal representative are required.)

法定代理人签字： 日期：

（注：如果受试者不能阅读该知情同意书时，则需一名独立见证人证明研究者已将知情同 意书的所有内容告知了受试者，独立见证人需签名和签署日期）

Signature of legal representative: date:

(note: if the subject is unable to read the informed consent form, an independent witness is required to prove that the researcher has informed the subject of all the contents of the informed consent form, and the independent witness is required to sign and sign the date)

研究者签名： 日期：

Signature of the researcher: date:
